# Properties, Advantages, and Prospects of Using Cobalt-Free Composites Based on Tungsten Carbide in Industry

**DOI:** 10.3390/ma18010129

**Published:** 2024-12-31

**Authors:** Sherzod Kurbanbekov, Yernat Kozhakhmetov, Mazhyn Skakov, Bekbolat Seitov, Madina Aidarova, Yerkezhan Tabiyeva

**Affiliations:** 1 Center of Excellence “VERITAS”, D. Serikbayev East Kazakhstan Technical University, Ust-Kamenogorsk 070004, Kazakhstan; ykozhakhmetov@ektu.kz (Y.K.); maidarova@ektu.kz (M.A.); etabieva@edu.ektu.kz (Y.T.); 2 The Research Institute “Natural Sciences, Nanotechnology and New Materials”, Khoja Akhmet Yassawi International Kazakh-Turkish University, Turkestan 161200, Kazakhstan; bekbolat.seitov@ayu.edu.kz; 3 National Nuclear Center of the Republic of Kazakhstan, Kurchatov 180010, Kazakhstan; skakov@nnc.kz

**Keywords:** cobalt-free metal alloy composites, tungsten carbide, nickel, iron, SPS

## Abstract

This paper reviews recent advances in the synthesis of cobalt-free high-strength tungsten carbide (WC) composites as sustainable alternatives to conventional WC-Co composites. Due to the high cost of cobalt, limited supply, and environmental concerns, researchers are exploring nickel, iron, ceramic binders, and nanocomposites to obtain similar or superior mechanical properties. Various synthesis methods such as powder metallurgy, encapsulation, 3D printing, and spark plasma sintering (SPS) are discussed, with SPS standing out for its effectiveness in densifying and preventing WC grain growth. The results show that cobalt-free composites exhibit high strength, wear and corrosion resistance, and harsh environment stability, making them viable competitors for WC-Co materials. The use of nickel and iron with SPS is shown to enable the development of environmentally friendly, cost-effective materials. It is emphasized that microstructural control and phase management during sintering are critical to improve a material’s properties. The application potential of these composites covers mechanical engineering, metallurgy, oil and gas, and aerospace, emphasizing their broad industrial relevance.

## 1. Introduction

As is known, composites based on metal alloys are complex materials consisting of two or more elements that combine to achieve unique physical and mechanical properties. In this category, tungsten carbide (WC) is one of the main components that ensures high hardness and durability of the composite. However, it is important to utilize suitable metallic elements as bonding phases to provide the required strength and fracture resistance.

Cobalt, often acting as a bonding phase, plays a crucial role in the mechanical performance of the composite. Its ability to form strong bonds with tungsten carbide particles increases the shear strength, which is especially important under intense mechanical loading conditions [1,2]. Due to this, WC-Co-based composites have a wide range of applications in various industries including metalworking, mining, and cutting tools.

Currently, WC is widely used in mechanical engineering, mining, and machine tool industries. Due to their high wear resistance, chemical resistance (corrosion resistance), melting point, and hardness, ceramics and tungsten carbide-based solid composites are used as wear-resistant materials [3,4].

At the same time, pure tungsten carbide has a low fracture resistance and a low bending strength; therefore, to reduce the sintering temperature and increase its physical and mechanical properties, plastic fusible metals (cobalt, nickel, iron, etc.) are introduced into the composition of ceramics [5,6].

However, in spite of a number of advantages, problems associated with the use of Co, such as price instability, toxicity, and the deterioration of some properties, have recently appeared in the use of WC-Co composite, which requires the fabrication of WC parts without a binder [7]. On the other hand, WC, or WC-based composites, have not been commercialized and produced on an industrial scale so far but have been used only to a limited extent for specialized applications such as high wear mechanical seals and high temperature electrical contacts [8]. As follows from the analysis of the available literature, there are two problems in WC development: the complete sealing of the sintered body and the achievement of high impact toughness. Kamolova I. O. et al. [9] provide a comprehensive summary of the current knowledge on the sintering behavior, microstructure, and mechanical properties of WC. They propose, to varying degrees, strategies for improving compaction, strengthening methods, and reference materials for material practitioners who wish to improve WC characteristics with greater reliability, marketing them for further widespread use and preparing the material in an environmentally friendly way via inexpensive production. Oskolova T. [10] showed that the production of high-density and high-performance WC is economically and technically feasible. The practically important properties of WC can be tuned by a judicious choice of chemical composition combined with careful consideration of the influence of production methods and processing parameters.

Along with this, WC-Co composites are known to be the strongest known sintered carbide composites, although they do not always meet the requirements for serviceability. Thus, in the total mass of amortized tools, the wear and breakage of carbide elements account for 80%. Therefore, it is believed that one of the promising directions for improving the properties of solid composites is the development of technologies to obtain and provide increased wear resistance while maintaining the necessary toughness. It is the totality of such properties of materials that provides durability of any tool made of them, perceived as high-intensity loads during cutting, stamping, rock drilling, etc. [11,12].

The most commonly used cemented carbides in practice consist of ceramic grains of tungsten carbide embedded in a metal matrix of cobalt. These WC-Co composites have an excellent combination of mechanical, physical, and chemical properties, making them suitable for a wide range of applications requiring high values of hardness, mechanical strength, and heat resistance [13,14].

Currently, there are many WC-Co based composites, differing in cobalt content, carbide particle size, the presence of various hardening and inhibiting additives, etc. Such composites always contain two obligatory components in their composition: a WC tungsten carbide, which acts as a “solid phase”, and a cobalt Co, which is used as a binder [15]. The combination of the excellent mechanical properties of tungsten carbide with the ductility and high fracture toughness of cobalt provides high hardness, strength, and wear resistance of WC-Co composites. Due to this, they are widely used as tool materials in metalworking and mining industries [16]. Moreover, many researchers believe that the most promising way to improve the performance characteristics of WC-Co-based solid composites is to create fine-grained microstructures or even nanostructures in them. This requires the use of fine and nanocrystalline powders of both tungsten carbide and cobalt, as well as special sintering procedures [17].

Cemented WC is commonly used for wear-resistant applications such as cutting tools and abrasives because of its extremely high hardness [18]. This hardness causes WC post-processing to become labor intensive and expensive. The bonding phase in the fabricated samples consists of an iron-based composite that has a lower melting point than cobalt, a common bonding agent in cemented WC. Cube-shaped WC samples with a low iron-based composite binder were printed by Ku N. et al. [19]; the effect of different processing conditions on the resulting density and microstructure of the material was investigated. Theoretical densities of up to 95% were achieved using this method. Artifacts indicative of the manufacturing process are present in the samples, and the problems associated with removing the processing history from the final microstructure are discussed.

This research focuses on improving WC-based composites by exploring alternatives to cobalt (Co) as a binder. While Co enhances toughness and strength, issues like price instability and toxicity drive the need for substitutes like iron (Fe) and nickel (Ni). The study orients at optimizing the WC composites’ properties and production methods, expanding their industrial applications while reducing the environmental and health impacts.

## 2. Problems of Cobalt Utilization in Traditional Composites: Resource, Environmental, and Economic Aspects

A detailed analysis of the studies performed shows that the use of cobalt in traditional composites faces a number of science-based challenges. First, cobalt is a rare element, which creates resource constraints as its reserves are concentrated in a limited number of countries, resulting in unstable supply chains. Second, the mining and processing of cobalt involve environmental risks, including pollution, as cobalt has toxic properties. Third, the high cost of mining and refining cobalt makes its use in composites uneconomical, especially with the increasing demand for this metal in modern technologies such as batteries and electronics [20,21,22,23,24].

This is because the raw materials used in production, i.e., WC and Co, are considered strategic by the European Union due to its supply risk [25,26]. In addition, the law on the regulation of the registration, evaluation, authorization, and restriction of chemicals of the European Union (EU-REACH programmer) and other studies classify cobalt and metal dust as potentially hazardous substances for living organisms, which further substantiates the need for solid-material alternatives to conventional WC-Co composites [27,28,29].

Thus, the need to find alternatives to conventional WC-Co composites is due to the high strategic importance of cobalt as a raw material whose reserves are mainly controlled by the People’s Republic of China [30]. In conditions of limited access to this metal, the risks of disruptions in its supply are increasing, which is especially relevant for the countries of the European Union, where cobalt is a key element not only for the production of WC-based tooling materials but also for the manufacture of batteries for electric vehicles. With the development of the electric vehicle market, cobalt consumption is growing rapidly, increasing its scarcity and price volatility. These factors emphasize the importance of developing alternative materials, such as nickel- and iron-based composites, which can not only replace cobalt in WC composites but also ensure supply stability and reduce the dependence on limited and expensive raw material sources [31,32,33,34]. Replacing Co without losing mechanical properties is possible, and the most promising materials for this purpose are iron (Fe) and nickel (Ni). These elements, used in various combinations and alloy systems, are capable of providing a high level of strength, heat resistance, and wear resistance. Modern technologies, such as the creation of multicomponent alloys or the use of protective coatings, make it possible to compensate for the disadvantages compared to cobalt and make Fe and Ni effective and practical alternatives [35]. Iron is a promising replacement for cobalt in WC hard metals due to its lower cost and toxicity. WC-FeCr shows improved creep resistance below 1000 °C, attributed to lower self-diffusivity and WC solubility compared to Co. However, above ~1140 °C, eutectic formation reduces its performance. These findings suggest that FeCr is suitable as a binder if operating temperatures remain below the eutectic point [36]. It should be noted that of the three elements, iron (Fe), nickel (Ni), and cobalt (Co) has traditionally been the most favored choice due to its outstanding properties such as high wettability and excellent adhesion to tungsten carbide. These characteristics make cobalt an important component in traditional WC-Co composites, where it not only contributes to the formation of a homogeneous microstructure but also provides strength, fracture resistance, and excellent mechanical properties [37].

However, there is thus some motivation for scientists and engineers to search for alternative metallic binders to cobalt that can provide comparable or even better mechanical performance at a lower cost and with less environmental impact. Therefore, the study of cobalt-free tungsten carbide-based composites without cobalt is becoming an important area in materials science, opening new horizons for the development of sustainable and cost-effective materials for industry.

Based on the above considerations, there is an urgent need to find substitutes for the traditional cobalt binder phase in the WC-based metal–ceramic hard coatings. This has prompted researchers to look for alternatives to cobalt and iron; nickel and some of their compounds are suggested as substitutes.

## 3. Alternative Bonding Materials for Tungsten Carbide-Based Composites

Cobalt-free WC composites are promising materials with significant advantages over the traditional WC-Co composites. Eliminating cobalt from the composition reduces health and environmental impacts, making such composites more sustainable and safer for humans. In addition, lower manufacturing costs due to the absence of cobalt make cobalt-free composites cost effective. They exhibit high mechanical properties including strength, wear resistance, and corrosion resistance, which enhance their application in industries such as mechanical engineering, metallurgy, and oil and gas [38]. Due to the technological flexibility provided by various synthesis methods, including powder metallurgy and 3D printing, cobalt-free composites can be adapted to specific operating conditions, including high temperatures and aggressive environments, which emphasize their relevance to modern manufacturing technologies. Along with this, cobalt-free WC-based metal alloy composites are promising materials for application in processes with high mechanical loads, temperatures, and wear resistance [39].

Growing concerns about the use of cobalt as a binder in WC-based hard composites have led to the investigation of iron alloys as alternatives. Studies have shown that replacing cobalt with Fe alloys under the strict control of carbon content significantly improves the mechanical properties. In addition to improving hardness and fracture toughness, the transverse tensile strength of WC-Fe composites shows significant potential for further improvement through microstructure improvement, such as reducing porosity during sintering [40,41,42,43].

Studies also show that cobalt-free solid composites containing nickel (Ni) and iron (Fe) as binders exhibit high levels of strength, wear resistance, and hardness. For example, the work of Qu et al. [44] shows that Ni-Fe-based composites can achieve compressive strengths of up to 3.5 GPa, while improving wear resistance by a factor of 1.5 compared to their cobalt counterparts. Furthermore, the results of a study by Wang et al. [45] emphasize that nickel and iron have good adhesion properties to tungsten carbide, providing a strong bond between WC particles, which are crucial for improving the mechanical properties of composites.

One of the key advantages of cobalt-free composites is their resistance to corrosive environments, making them attractive for corrosive applications. Studies confirm that nickel provides high corrosion resistance in chemically aggressive environments such as acids and alkalis [46]. Iron, in turn, improves mechanical properties and contributes to the hardness of the material. The work of Xu et al. [47] showed that the combination of nickel and iron can produce property-balanced composites with good strength and wear resistance, which is a significant advance over traditional cobalt composites.

Current research also shows the high potential of cobalt-free composites for applications in various industries including engineering, oil and gas, metallurgy, and aerospace. For example, research [48] demonstrates that the application of the SPS technology can achieve high composite densities with minimal grain growth, which is crucial for maintaining the wear resistance of the material. In addition, Peter et al. [49] emphasize that Ni-Fe-based composites are not only able to compete with their cobalt-based counterparts but also open new horizons for the creation of safer and more cost-effective materials.

Apparently, the development and study of cobalt-free composites based on tungsten carbide with nickel and iron are actual directions of scientific research that can significantly influence the future of materials science and the industrial application of solid composites.

**Figure 1:**
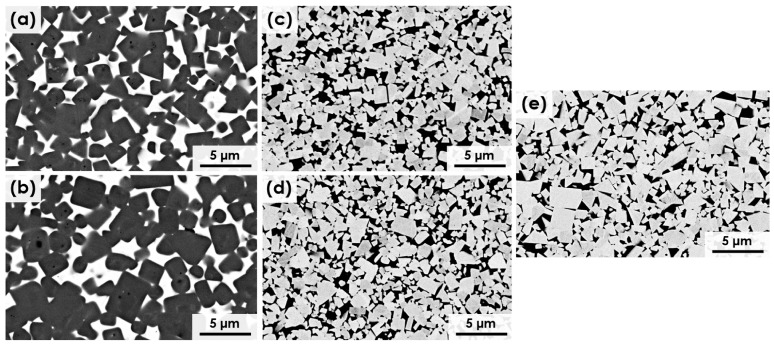
SEM micrographs (BSE mode) of the microstructure of sintered materials: (**a**) Ti(C,N)-FeNi, (**b**) Ti(C,N)-FeNiCr, (**c**) WC-FeNi, (**d**) WC-FeNiCr, and (**e**) WC-Co [58].

Nickel is known to have sufficient mechanical strength, good corrosion resistance, and relatively low cost. Therefore, it may be one of the most promising solutions for replacing cobalt in the WC composites. When nickel is added, the WC-Ni composites can retain high hardness and wear resistance, although these values may be lower compared with WC-Co. The use of nickel not only reduces the material cost but also the environmental load. Indeed, as follows from the results of [50,51], the replacement or displacement of Co by Ni improved the corrosion resistance as well as the wear resistance of WC-based metal alloy composites in aggressive environments. Fe and Ni as alternative binders in WC-(Fe-Ni) composites provide significant advantages. Ni is more suitable for applications requiring corrosion and oxidation resistance, mainly in tribological applications [52,53,54,55]. Studies by Zhang Z. et al. and Frazier W. E. [56,57] were carried out on the preparation of WC-(Fe-Ni)-based solid composites using iron and nickel, as substitutes in whole or in part. They showed that a WC-(Fe-Ni) composite can have better mechanical and microstructural properties compared to WC-Co composites. Specifically, the formation of Fe-Ni-based intermetallics, such as (Fe,Ni)_3_W_3_C, enhances grain refinement, grain boundary strength, and thermal stability compared to Co-based binders. This leads to reduced grain growth during sintering, greater resistance to cracking, and mitigates brittle η-phase formation. As a result, Fe-Ni binders promote a more uniform WC grain distribution, improving toughness and wear resistance. In addition, WC-Ni-Fe components are mutually inert, without chemical interaction during sintering, which can preserve the shape and the high strength of the composite. It follows from the above that Figure 1 depicts the microstructure of the five studied materials. The ceramic grains are visible as the dark phase in Figure 1a,b and as the bright phase in Figure 1c–e, showing a uniform distribution within the metallic matrix. Additionally, all materials demonstrate full densification, reflecting optimal microstructural uniformity and consolidation [58].

However, a significant amount of Fe can reduce the optimal range of free carbon (“carbon window”) and thus favor the formation of η-phase. In such materials, the η-phase is usually represented by the compounds of the M_6_C or M_12_C type, where M denotes a metal such as tungsten (W) or cobalt (Co). It is formed as a result of reactions between the metallic elements and carbon in the system. However, the formation of the η-phase must be carefully controlled, since an excessive amount can lead to a decrease in ductility and the deterioration of the overall mechanical properties of the material. The optimal balance between the WC phases, the metallic binder, and the η-phase is the key to creating high-performance hard alloys. An important aspect is the effect of the carbon content on the phase transformations and adhesion; the optimal amount prevents the formation of brittle η-phases and promotes the formation of a liquid phase during sintering, which improves the compaction, minimizes the porosity, and reduces the residual stresses. This approach allows for creating composites with excellent hardness, wear resistance, and strength [59].

Aluminum is less commonly used in WC composites and is added to reduce the overall density of the material and to improve oxidation resistance at high temperatures [60]. Aluminum can contribute to hardness, but excessive amounts can reduce the ductility of composites [61]. However, when combined with other elements such as molybdenum or copper, aluminum can help to improve the overall balance of properties. Silicon is added to WC composites to improve thermal stability and wear resistance [62]. It also helps to improve corrosion resistance by reducing the tendency to oxidize at high temperatures. However, at high concentrations, silicon can increase the brittleness of composites, which limits its application.

According to Kim J. et al. [63], the interfacial bonding properties of WC/Co and WC/ CoNiFe were studied based on the aforementioned physical and mechanical properties. WC/Co and WC/CoNiFe materials were synthesized using conventional powder metallurgy, and the modeling results were verified via microstructure analysis, electron function gradient method, and mechanical property evaluation. The main findings of the study are as follows: The binding energies at the WC/Co and WC/CoNiFe interfaces were calculated according to the first principle, and the binding energy of Co with WC is slightly higher than that of CoNiFe with WC. This suggests that replacing Fe and Ni with part of Co weakens the bonding properties at the WC/Co interface, but the effect is negligible.

The atomic bond strength at the WC/Co and WC/CoNiFe interfaces is mainly determined by the contribution of the d-electron orbitals of the atoms. According to the difference in charge density and the density of states, the atomic bond strength is W-Fe > W-Co > W-Ni. Fe atoms play a major role in the strength of a WC/CoNiFe interface.

The porosity of WC/CoNiFe carbide is 0.78%, which is much lower than that of a WC/Co material. The WC/Co material has a non-uniform pore distribution. The highest average hardness and the lowest average hardness of WC/Co carbide are 1320 HV and 1182 HV in samples with non-uniform hardness distribution. The hardness of WC/CoNiFe carbide is relatively more uniform, with the highest value of 1192 HV [64].

The densification mechanism at the first heating stage is related to the plastic flow process, the intensity of which is limited by the diffusive creep rate of cobalt. Increasing the concentration of free carbon in the composition of the initial WC-10Co mixture does not affect the intensity of the densification process and, consequently, the activation energy of EIPS in the region of “average” heating temperatures. The activation energy of isothermal and non-isothermal sintering in the region of “medium” temperatures is close to the activation energy of grain boundary diffusion in cobalt. In the region of “high” temperatures, the activation energy of non-isothermal sintering monotonically decreases with increasing concentration of free carbon in WC-10Co carbide. It was shown by the X-ray phase analysis that the reason for the decrease in the activation energy of EIPS may be due to a change in the concentration of tungsten and carbon atoms in cobalt [65].

In addition to the above studies conducted to completely replace Co with alternative bonding components, a study was also conducted to partially replace Co with Ni and Fe [66].

**Figure 2:**
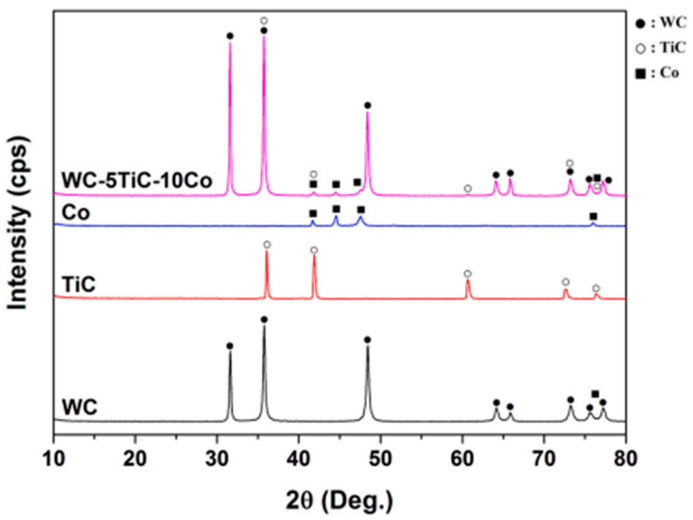
Phase composition of the original mechanochemically synthesized WC, TiC powders, branded Co powder, and their compositions of WC-5TiC-10Co after mixing/grinding [68].

The effect of composition on carbide formation in the WC-M (M = Fe/Ni/Cr) system was studied with three compositions containing 10 wt.% metallic binder [67]. Replacing half of the Fe binder with Ni stabilized the formation of austenite Fe (γ). Adding Cr to the binder led to Cr_2_C carbide formation, while reducing Cr content resulted in M_6_C carbide instead. To eliminate M_6_C, 3.6 wt.% of excess carbon was added. These results were analyzed based on phase formation, binder composition, and carbon content. According to Buravlev, I. Y. et al. [68], a method for obtaining a WC-5TiC-10Co carbide with a density equal to 100% of the theoretical value, a hardness of 1484 HV, and a bending strength of 1924 MPa is presented. The high quality of the product is provided by the original research method based on the mechanochemical synthesis of WC and TiC powders using available precursors and via the implementation of high-speed SPS compacting of the obtained powder with achievement of high packing degree with minimum grain growth at temperature 1200 °C and processing time not more than 13 min. The experimental data on the compaction dynamics, phase composition, morphology, and mechanical properties of WC-TiC-10 Co using SPS in the temperature range from 1000 to 1200 °C are presented. As can be seen from the data, Figure 2 shows the XRD results for WC and TiC powders from mechanochemical synthesis and for Co powder from mechanical mixing/grinding in a planetary mill. The results confirm that the specified mixing/grinding parameters do not produce additional phases. The XRD curve for WC-5TiC-10Co reveals increased WC peak intensity, attributed to the deagglomeration of large WC particles [68].

**Figure 3:**
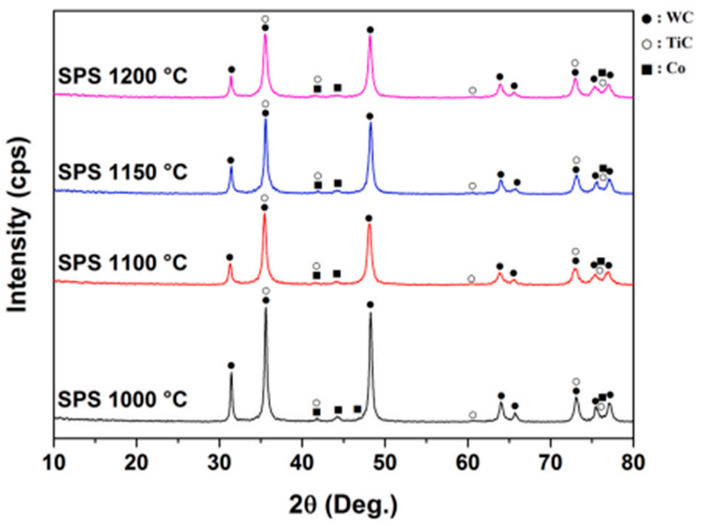
Phase composition of WC-5TiC-10Co alloys obtained through SPS consolidation at different sintering temperatures (1000 °C, 1100 °C, 1150 °C, and 1200 °C) [68].

It is noteworthy that Figure 3 shows that the XRD analysis confirms that the phase composition of the SPS-consolidated samples remains stable at sintering temperatures ranging from 1000 to 1200 °C. Elevated sintering temperatures enhance the distribution of the cobalt binder in the matrix, as evidenced by the decreased intensity and peak broadening of Co. The WC phases exhibit reduced intensity, particularly in samples sintered at 1100–1200 °C, due to the formation of a (Ti,W)C solid solution along TiC grain boundaries, resulting from metal substitution during high-temperature diffusion in SPS [68].

**Figure 4:**
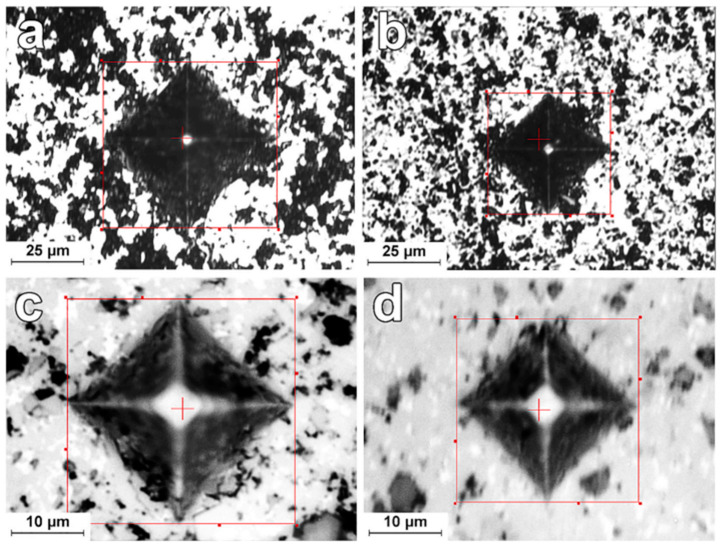
Microphotographs of prints of the microhardness tester indenter at load HV0.5 on the surface of investigated WC-5TiC-10Co samples obtained through SPS consolidation at different sintering temperatures: (**a**) 1000 °C (×40); (**b**) 1100 °C (×40); (**c**) 1150 °C (×100); (**d**) 1200 °C (×100) [68].

Buravlev, I. Y. et al. [68] report surface images showing the indenter prints from the microhardness tester under a load of HV0.5, indicating an absence of propensity for cracking in WC-5TiC-10Co alloys obtained via SPS under varying sintering temperatures, as shown in Figure 4.

**Figure 5:**
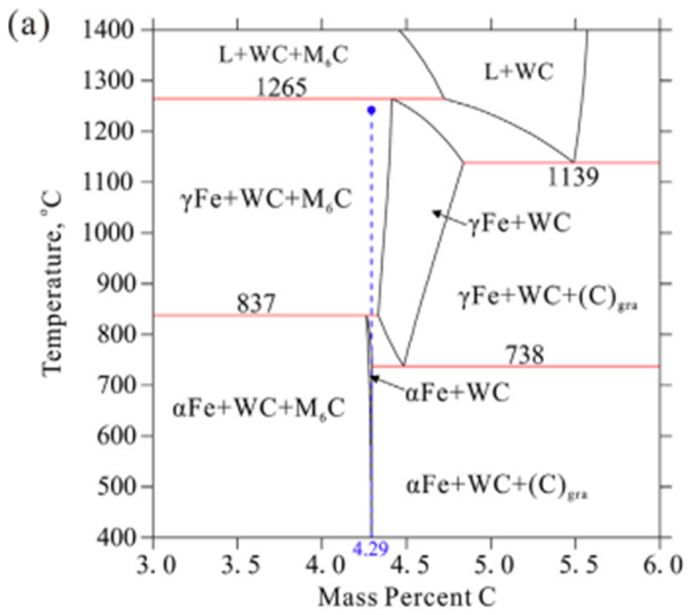

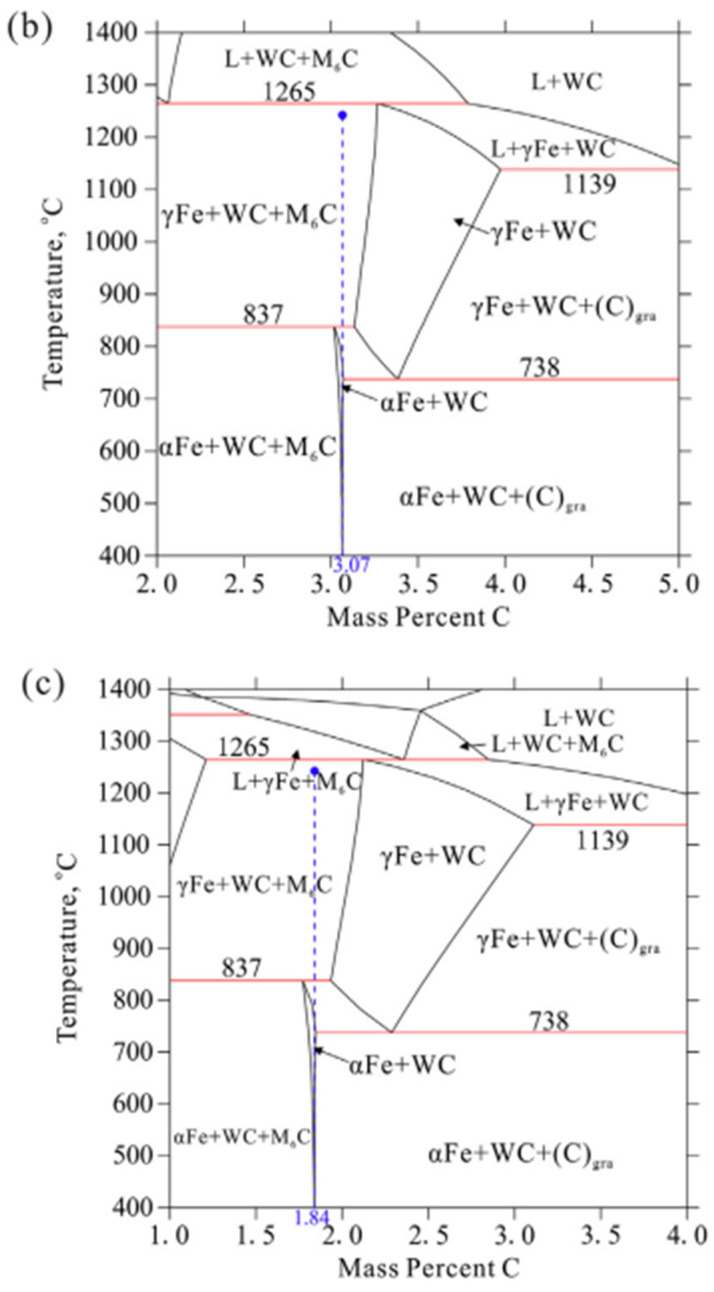
Thermodynamic-calculated phase equilibria close to the sintering region of (**a**) WCe30 wt % Fe, (**b**) WCe50 wt % Fe, and (**c**) WCe70 wt % Fe. The vertical dashed line indicates the carbon content of the samples prepared in the present work [69].

Pan et al. [69] confirmed the formation of η- phase in composites of WC-30% wt. Fe, WC-50% wt. Fe, and WC-70% wt. Fe. From the presented data, it follows that Figure 5 presents the calculated phase diagrams (vertical sections) of W-Ce-Fe alloys with varying Fe contents. The carbon content of the composites is optimized to lie within the two-phase sintering region, consisting of WC and α-Fe phases, to ensure phase stability and favorable microstructural properties.

The use of cobalt-free WC-based composites, in which cobalt is replaced by nickel and iron, represents an important technological step in the development of sustainable and cost-effective materials [70]. These composites retain key mechanical properties such as high hardness and wear resistance, while exhibiting improved corrosion and crack resistance due to the uniform distribution of nickel and iron in the WC matrix. Not only are cobalt-free composites less expensive due to the substitution of scarce and expensive cobalt, but they are also more environmentally friendly, thus reducing environmental impact. In addition, the stability of their supply reduces the dependence on strategically limited resources, making such materials more attractive for use in a variety of high-tech industries, including mechanical engineering, aerospace, and energy.

## 4. Technologies for Synthesis of WC Composites

Synthesis technologies for WC composites include various methods aimed at optimizing the mechanical properties and performance of the materials. The most common technologies are powder metallurgy, SPS, hot isostatic pressing (HIP), and 3D printing.

The synthesis technology is directly related to the composite as it determines its microstructure, density, and mechanical properties. For example, powder metallurgy and spark plasma sintering (SPS) methods allow for the controlling of grain size and density, which significantly affects the hardness and wear resistance. Hot isostatic pressing (HIP) improves flexural strength and impact toughness by eliminating internal defects. Three-dimensional printing provides the ability to create complex shapes and optimize the composition of the binder phase, including replacing Co with alternative metals such as Fe and Ni. These technologies not only improve the properties of WC composites but also allow them to be adapted to specific industrial requirements.

To date, there are many technologies for the synthesis of cobalt-free solid WC composites. For example, according to Kazantseva N. et al. [71] on the partial substitution of Co by Ni and Fe, WC/Co and WC/CoNiFe materials were prepared using conventional powder metallurgy. WC, Co, Ni, and Fe powders (with a mass ratio of 6:2:1:1:1) as well as WC and Co powders (with a mass ratio of 6:4) were mixed using a Retch GmbH planetary ball mill for 1 h at a rotational speed of 200 rpm, and then the mixed powders were pressed into cylindrical billets with a diameter of 3 mm and a height of 12 mm using a WDW-300 universal testing machine at an instantaneous pressure of 1000 MPa. Kelvin probe force microscopy (KPFM) results showed that the interface bond strength of conventional WC/Co carbide is consistent with the results of first-principles calculations and slightly higher than that of WC/CoNiFe carbide, but the addition of Ni and Fe atoms does not weaken the interface strength significantly.

The following encapsulation technology is a technique in which WC particles are coated with a layer of binder material [72]. This process favors a more uniform distribution of the binder on the surface of the carbide particles, which improves the quality and homogeneity of the composite [73]. In addition, encapsulation provides better wetting and fluidity of the material during the sintering process, which helps to reduce porosity and increase mechanical strength [74]. This method is particularly effective for the creation of composites with fine-grained structures, which have increased hardness and wear resistance [75]. However, encapsulation requires the careful selection of binder and shell materials to prevent structure failure at high temperatures [76]. The encapsulation process can lead to non-uniform coating of carbide particles, which deteriorates the mechanical properties of the composite and its wear resistance Additive manufacturing technologies such as 3D printing open new horizons in the synthesis of cobalt-free composites [77]. The layer-by-layer deposition of powder materials and their sintering allow for the fabrication of products with precise geometries while reducing material and manufacturing costs [78]. However, the disadvantage is that 3D printing is slow and less productive for large-scale industrial production, which limits its application.

There is also a method of OPS-oscillatory pressure sintering. This method can improve compaction and reduce the grain size of the material and was applied in [79]. This work showed that the OPS-sintered composite exhibited solid-phase sintering at low temperatures (<1240 °C) and liquid-phase sintering at higher temperatures (>1240 °C). In both temperature ranges, the OPS-sintered samples have higher densities.

The sintering of WC-20Ti cemented carbide was carried out using the laser powder bed fusion (LPBF) method, in which a powder mixture consisting of WC and 20 wt.% Ti was fused layer by layer under laser irradiation. The optimum printing parameters included a laser power of 175 W and a scanning speed of 600 mm s^−1^, ensuring high density and uniformity of the microstructure. During the process, the laser energy caused the melting of the powder particles, promoting their dense sintering and the formation of homogeneous phases, including WC, W_2_C, and TiC. Due to the controlled heating and cooling conditions, an average grain size of about 5.02 μm was achieved, which provided the improved mechanical and corrosion properties of the material [80].

Aluminum (Al), copper (Cu), zinc (Zn), titanium (Ti), molybdenum (Mo), and zirconium (Zr) are less suitable than Fe and Ni as the binders for metal cutting tools due to limitations such as low hardness (Al), insufficient strength (Cu), low melting point and brittleness (Zn), reactivity and low wear resistance (Ti), high cost (Mo), and brittleness (Zr). Fe and Ni offer advantages such as economic availability, versatility in alloying, corrosion and oxidation resistance, and lower environmental impact. Cobalt (Co) offers high wear and heat resistance, but Fe and Ni provide a more cost-effective and environmentally friendly solution. The alloying additives such as Al (up to 2%), Cu (1–5%), Si (1–3%), Ti (1–4%), and Mo (3–10%) optimize properties such as the corrosion resistance, strength, thermal conductivity, wear, and heat resistance. A proper combination enhances composite properties for their specific applications [81].

WC-Co hard metal samples were fabricated using WC-Co nanopowder with selective laser melting (SLM) and fused deposition modeling (FDM). Carbon loss during SLM led to a lower relative density and hardness, while FDM produced more stable WC phase cemented carbide with a higher density due to optimized filament packing. The sintered FDM samples achieved a relative density of 96.3%, a hardness of HRA 89.06, and a carbon content of 5.47–5.52 wt.% [82].

A WC-Co composite was produced by material extrusion 3D printing, overcoming casting challenges due to high melting points. A 54 vol% solid content paste was optimized for dispersant, solvent, and binder composition, achieving homogeneity through a binder coating. Sintering at 1400 °C in vacuum yielded a relative density of 99%, with hardness and fracture toughness measured at 16.5 GPa and 16.5 MPa·m^1/2^, comparable to powder metallurgy WC-Co. This method improves design flexibility, production time, and cost efficiency, which are especially beneficial for new product development [83].

Liquid ink printing followed by sintering was used to fabricate WC-Co microlattices and cutting tools, with WC_-x_Co (x = 0.5–20 wt.%) microstructures examined at varying Co ratios and sintering temperatures. Microlattices with 0.5–5 wt.% Co showed residual porosity due to incomplete densification, while fully dense struts were achieved with 10 wt.% Co at 1450 °C. WC-10Co lattices, modeled by finite-element methods, demonstrated elastic support through a 0/90° cross-ply structure, while Cu infiltration at 1300 °C formed WC-Cu composites with high thermal conductivity (140 ± 7 W/(m·°C)). The WC-Cu structure exhibited ductile compression behavior, contrasting the brittleness of WC-Co lattices. A 3D-printed WC-Cu tool exhibited lower temperatures under laser heating than a uniform WC-Co tool due to its enhanced internal architecture [84].

Three-dimensional gel-printing (3DGP) is an innovative method for creating 3D components by layering and gelating metal slurry. Using WC-20Co slurries with 47–56 vol.% solid loading, components were formed via 3DGP and sintered in a vacuum furnace. The slurries’ fluidity and shear-thinning behavior supported the 3DGP process, with nozzle diameter and filling rate affecting surface quality and dimensional accuracy. Optimal samples showed good shape retention, uniform microstructure, a density of 13.55 g/cm^3^, a hardness of HRA 87.7, and a transverse rupture strength of 2612.8 MPa, making 3DGP effective for the near-net shaping of complex WC-20Co parts [85,86,87].

**Figure 6:**
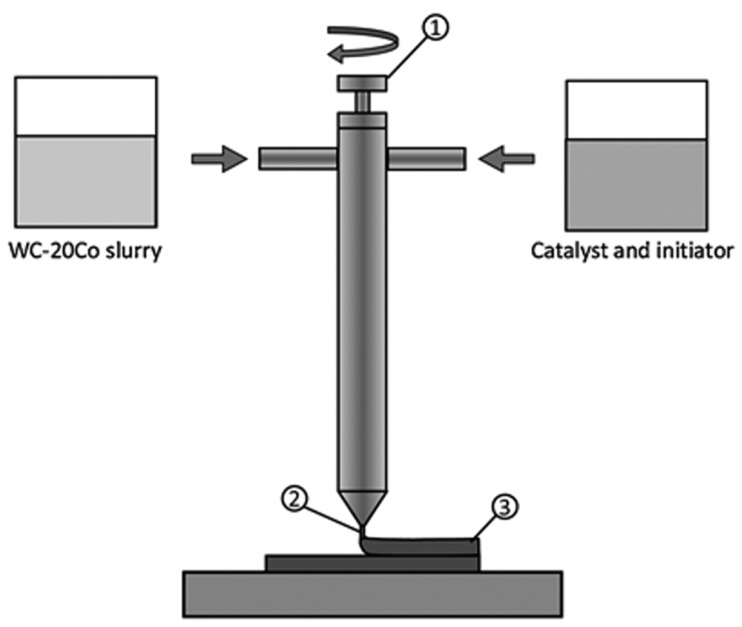
Schematic diagram of 3DGP, (1) screw extruder, (2) nozzle, and (3) green body [88].

Aramian A. et al. [88]’s 3DGP technique involves a slurry of powder and organic monomers that is fed into a screw extruder with compressed air pressure, where an initiator and catalyst are added, mixed, and extruded layer by layer onto the build platform, which is shown in Figure 6. The organic monomer quickly polymerizes, fixing the solid powder in place and forming a green body.

Binder jet additive manufacturing (BJAM) is an additive technology in which metal powders are bonded with a liquid binder and then sintered to form high-strength parts. In the BJAM process, the powder is distributed on a substrate and bound with liquid binders to produce a ‘green’ part, which then goes through a binder removal and sintering step to form a metal part. The sintering temperature for WC-Co is typically above 1400 °C. The fabricated parts are often highly porous and may be impregnated with a metal such as cobalt to improve properties, making it difficult to accurately control the Co content of the sample [89,90].

**Figure 7:**
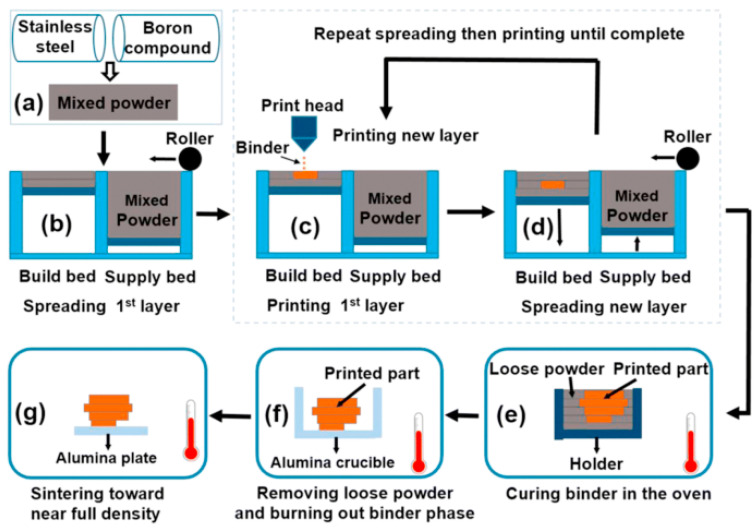
Schematic diagram of the BJAM process [91]. The process generally involves the following steps: (**a**) Mixed powder, (**b**) spreading the mixed powder and printing, (**c**) depositing or printing the binder phase onto the specific areas of the mixed powder layer intended for consolidation, (**d**) repeating steps (**b**) and (**c**) until the part is fully formed, (**e**) curing the binder phase, (**f**) removing excess powder, burning out the binder phase, and partially sintering the printed material, and (**g**) infiltrating with a secondary metal that has a lower melting point.

Do T. et al. [91]’s WC-Co alloy was obtained by 3D printing via material extrusion. A paste with a high solid content was prepared, and the paste composition was improved for rheological characteristics depending on the dispersant, solvent, and binder content, which is shown in Figure 7. The BJAM system operates with two distinct powder beds: one dedicated to building the part and the other supplying the powder. The build bed is positioned slightly lower by one layer to make space for a new powder layer, which is spread from the supply bed to the build bed using a roller (in larger systems, a hopper may also be utilized).

Kim et al. [92] investigated WC-based cermets with Ni as a bonding phase. They were able to fabricate WC-Ni-based cermets with a density of 98% using high-frequency induction heating by sintering (HFIHS). The sample had a WC grain size of 300 nm after sintering. The measured hardness values were significantly higher than the traditionally sintered WC-Co and WC-Ni systems without any noticeable decrease in fracture toughness.

Fused filament fabrication (FFF) is an additive manufacturing technology similar to 3DGP but uses a powder to prepare the filament instead of a powder slurry as the printing material. The filament preparation involves mixing the powder with a main binder and a backbone, followed by extrusion using a high-pressure capillary rheometer. During the printing, the filament is deposited on a printing platform to form a green part. After the printing, a deboning process, including solvent deboning followed by thermal deboning, is performed before sintering the part [93].

**Figure 8:**
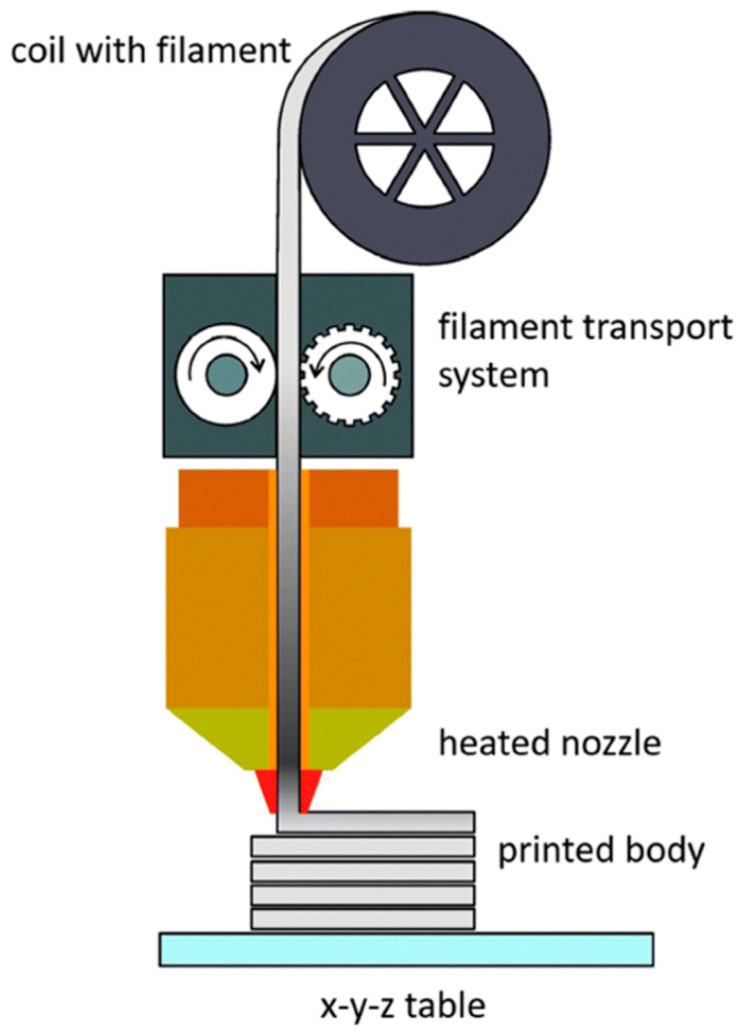
Schematic diagram of FFF [93].

Kukla C. et al. [93] described that this basic principle allows for the production of complex parts without a molding tool, except for a die with a simple geometry that is usually circular. Depending on the type of extruder used, additive manufacturing by material extrusion can be classified into different types, which will be described in the next section, and is shown schematically in Figure 8.

Selective electron beam melting (SEBM) is limited to conductive materials because it requires electrical conductivity. The main difference is in the heat sources; SEBM uses a steerable electron beam with a higher scanning speed, requiring a vacuum, while selective laser melting (SLM) uses a laser and argon shielding. The electron beam penetrates deeper into the material than a laser, and the temperature is higher in SEBM. SEBM also requires fewer support structures because the sintered powder supports the product, giving SEBM more geometric freedom compared to SLM. Thus, the differences between these technologies are presented in Table 1 [94].

In the summary by [94], the five additive manufacturing (AM) discussed processes can be divided into two categories: selective melting processes and shaping-deboning-sintering (SDS) processes. SLM and SEBM use a heat source to melt powder and form parts, offering a simple, one-step molding process but often requiring post-processing to address stress and defects. The SDS processes, including BJAM, 3DGP, and FFF, involve forming a green part using organic binders, followed by sintering. These processes are more complex than selective melting as they require a powder spreading step, which necessitates good powder flowability in SLM, SEBM, and BJAM. However, 3DGP and FFF use powders in a slurry or filament form, eliminating the need for powder flowability. SEBM’s application is limited due to its high equipment cost, while SLM faces issues with uneven microstructure, carbon loss, and Co evaporation.

A novel tungsten steel was prepared using selective laser melting (SLM) and hot isotropic pressing (HIPing). Initially, a powder mixture of maraging steel and WC was used in SLM to produce a net-shaped composite with γ (Fe, Ni) (austenite) and WC phases. After HIPing, the microstructure evolved into an α-Fe matrix (ferrite and martensite) with Fe(Ni)-W-C η-phase precipitates in sub-micro and nano scales, located at grain boundaries and within grains. The process formed a stable, graded multi-scaled structure at the WC-matrix interfaces. HIPing improved mechanical properties, increasing the ultimate tensile strength from 980 MPa to 1470 MPa and the yield strength from 561 MPa to 1025 MPa, with elongation slightly reduced from 7% to 6% [95].

Cemented WC-Co is widely used in cutting, machining, and mining tools due to WC’s hardness and Co’s toughness. However, Co is a critical material with carcinogenic concerns, prompting research into alternative binders like nanostructured FeNiZr. The consolidation of WC-FeNiZr powders using field-assisted sintering and hot isostatic pressing produced dense samples with superior hardness (16 GPa) and toughness (13 MPa·m^1/2^). Electron backscatter diffraction and transmission electron microscopy reveal the structure–property relationships, demonstrating FeNiZr’s potential as a non-toxic Co substitute [96].

Fudger S. et al. [97] compared the microstructural development and mechanical properties of tungsten heavy alloys (WHAs) with FeNiCoCrMn high-entropy alloy (HEA) and conventional FeNi binders. Both WHAs were fabricated via hot isostatic pressing at 1450 °C in argon. Scanning electron microscopy showed uniform, refined microstructures for both binders, with HEA forming a skeletal network around tungsten grains. HEA binder increased micro Vickers hardness by 42% and enhanced the hardness of the WHA. However, WHA with HEA exhibited faster strain hardening and premature failure, resulting in lower ultimate strength and reduced ductility compared to the Fe-Ni binder.

This study examined the effects of NbC on densification, grain growth, and properties of WC-10AISI304 hard metals sintered via pseudo hot isostatic pressing at 1300 °C under vacuum and 20 MPa pressure. Adding 2 wt.% graphite reduced η-phase formation, while varying NbC content (1–5 wt.%) influenced the microstructure and other properties. NbC addition refined WC grains and reduced η-phase formation, with 2 wt.% NbC achieving the best results: the highest hardness (1820 kg/mm^2^) and a moderate fracture toughness (7.7 MPa·m^1/2^). Excess NbC (5 wt.%) led to coarse (Nb, W)C grains and decreased hardness [98].

High-density WC-FeNi cermet composites were fabricated via liquid-phase spark plasma sintering (SPS/FAST) using micron-sized powders of WC, Fe, Ni, and C. The in situ formation of an FeNi alloy binder was enabled via a eutectic reaction, with carbon stabilizing the binder phase. The composite achieved a theoretical density of 99% with a microstructure of rounded WC grains averaging 10.5 μm in size. It exhibited a maximum hardness of 16.1 GPa, offering a fast and cost-effective method for producing hard metals [99].

Taking the above into account, it should be noted that the technologies of synthesis of WC-based composites continue to develop, aiming to improve their mechanical and operational properties. Techniques such as powder metallurgy, encapsulation, SPS, and hot isostatic pressing produce composites with high hardness, density, and wear resistance. Alternative approaches, such as partial replacement of cobalt with nickel and iron, as well as the introduction of additive technologies and sintering, open new perspectives in the production of environmentally friendly and cost-effective materials. However, despite the successes achieved, further research and development is needed to scale up these technologies and their application in industry.

## 5. SPS Synthesis for WC Sintering

The SPS method is a transformative technology for manufacturing and advanced material research. Fifth-generation SPS systems provide enhanced functionality, reproducibility, and cost efficiency, enabling the production of larger, high-quality components for applications in electronics, automotive, cutting tools, fine ceramics, clean energy, and biomaterials. For instance, nano-WC powder is densified with a nano-grain structure for optical uses [100,101].

Invented in Japan, SPS has become a global innovation, with over 600 units in Japan and 1100–1300 worldwide as of 2020, facilitating advanced material fabrication across industries in Europe, the USA, and Asia.

**Figure 9:**
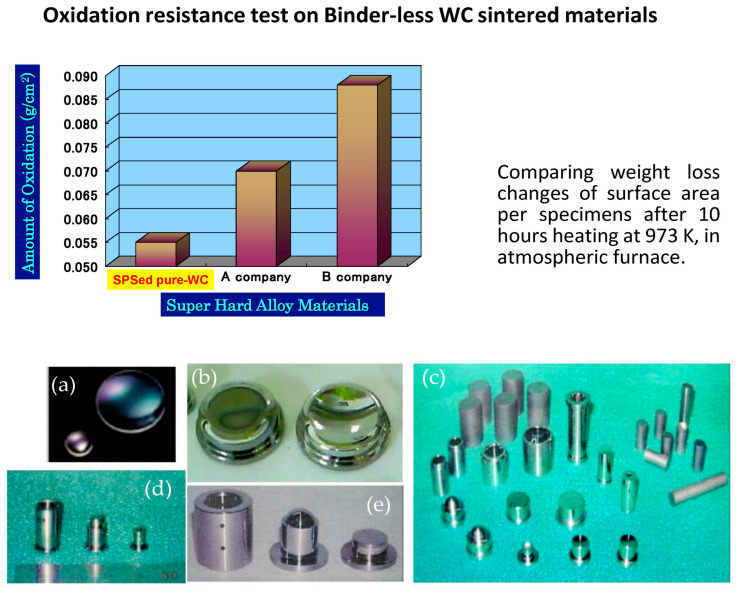
Examples of pure WC aspheric glass lens mold materials s: (**a**) formed small and large aspheric glass lens; (**b**) large-size aspheric glass lens molding die; (**c**) various SPSed compacts for glass lens die material and finished parts; (**d**) finished glass lens sleeve die and punches for mobile phone applications; and (**e**) finished glass lens sleeve die and punches for digital camera applications [102].

In optics, an SPSed aspheric glass lens mold is made from binderless pure WC (HV2600) without additives. Starting with powders below 200 nm, the material is sintered into a nanostructured fine grain, achieving a mirror surface roughness of R_a_ 2–6 nm after post-processing. This mold, consisting of an upper punch, lower punch, and sleeve die, demonstrates SPS’s advantages including additive-free sintering, finer grains, and 30–60% better oxidation resistance than conventional methods after a 10 h test at 700 °C, as shown in Figure 9.

**Figure 10:**
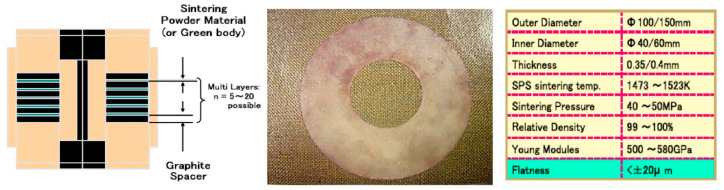
WC/Co-sintered very thin plate with a diamond-dicing blade [102].

A practical application of SPSed mass production is in the cutting tools and wear-resistant materials industry. Factories run 2–3 shifts daily, producing 15–20 plates per batch. Co-bonded WC dicing blades (100–150 mm diameter, 0.35–0.4 mm thickness) achieve flatness within ±20 μm with minimal residual stresses, as shown in Figure 10. SPS processing at 1200–1250 °C results in WC/Co plates with 99–100% density and a Young’s moduli of 500–580 GPa, eliminating the need for grinding and significantly reducing costs. Additionally, SPS facilitates the production of Cu-based dicing blades under 0.3 mm width. Its efficiency in near-net and net-shape forming has led to its widespread adoption by several companies for large-scale production.

WC/Co and WC/Ni cemented carbides are widely used as high wear-resistant materials in press-stamping dies and cutting tools. While conventional sintering requires extended processing times, new automated SPS systems enable the rapid fabrication of these materials, significantly reducing production time, as shown in Table 2. SPS technology’s rapid sintering capabilities enhance efficiency and maintain high material quality. The typical mechanical properties of SPSed WC/Co alloys and binderless nano-grain WC products demonstrate superior performance, highlighting the advancements in this technology.

Tokita M. explores the history, fundamentals, and industrial applications of SPS, emphasizing its role in ceramics, nanocrystalline materials, FGMs, and wear-resistant hard materials. The global adoption of SPS has surged in the past decade, with increased research and patents, highlighting its unique ability to achieve high-density sintering through energy concentration in conductive areas. This capability supports cost-effective production and expanded applications across industries such as automotive, electronics, aerospace, clean energy, and optical manufacturing. SPS’s key advantage lies in controlling nano-microstructures, with proven success in solid-phase sintering of pure nano-WC powder to near-full density, commercialized for glass lens molds in the optical sector [102].

A review of the available literature shows that SPS is a promising synthesis method for creating WC-based hard composites. SPS promotes uniform phase distribution in the material, which enhances its crack and wear resistance. Due to the fast heating rate and reduced holding time at high temperatures, this method minimizes the formation of defects such as porosity and unwanted phases. Therefore, the use of SPS for the synthesis of WC composites ensures high strength, microhardness, and structural stability, making this method particularly effective for producing high-performance and durable hard materials for various industrial applications [103,104].

The SPS method, as electro-discharge sintering [105], is a promising technology for the consolidation of powder materials. This method, also known as field-assisted sintering technology (FAST), plasma assisted sintering (PAS), electro consolidation, high-energy high-rate processing (HEHR), or electric discharge compaction (EDC) [106], is a new sintering technique used to produce nanostructured, composite and gradient materials. The SPS method was applied in [107,108] to obtain Ti-Al-Nb-based metalohydrides. SPS is a technology that consists of passing an electric current directly through the mound and the work piece to be pressed, rather than through an external heater. This results in a “spark plasma effect” and provides very sharp heating and consequently short cycle times. This fact makes it possible to suppress grain growth and obtain an equilibrium state. This opens up the possibility of creating new materials with previously inaccessible compositions and properties, materials with submicron or nanoscale grains, and composite materials with unique or unusual compositions [109,110,111].

These features of SPS technology include the precise control of the high-speed heating process, a controlled hydraulic pressure application system, and a high level of automation in modern SPS systems, which are equipped with an efficient feedback system, pyrometers for temperature control, built-in dilatometers for online shrinkage monitoring, and the ability to measure powder shrinkage rates [112].

Modern systems for SPS allow us to change all important process parameters (heating rate, temperature, and sintering heating process). This makes the SPS method an extremely effective way to control the microstructure parameters of ceramics- and tungsten carbide-based hard composites [113].

**Figure 11:**
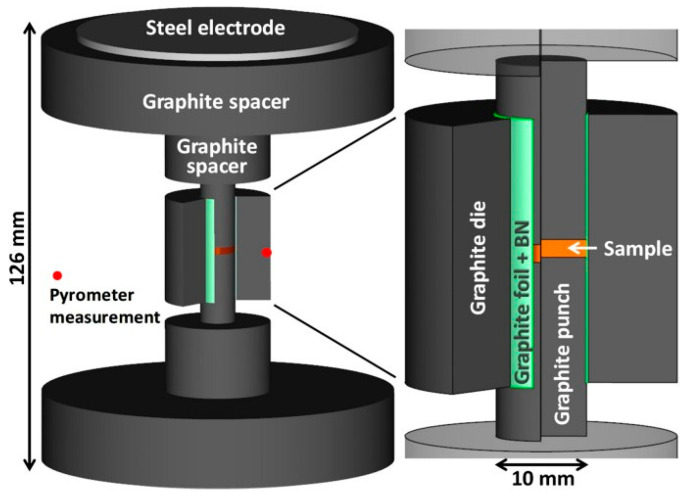
Structure of SPS synthesis [114].

In this work, a SPS machine (SPSS DR.SINTER Fuji Electronics model 5015, manufactured in Osaki, Japan) was used. In the net-shaped flash spark plasma sintering (NSFSPS) configuration, unlike traditional SPS, two graphite foils are inserted with a 0.4 mm gap, and the inner die surface is coated with an insulating boron nitride layer to concentrate the current on the sample, as shown in Figure 11. Powders with different electrical conductivities were used to test the method: Ni, Al_2_O_3_, and 3Y-ZrO_2_. The SPS process has a significant impact on the sintering of WC composites due to the possibility of rapid heating and precise temperature control, which helps to minimize grain growth and increase the density of the material. In the NSFSPS modification, the use of graphite foils and a boron nitride layer on the inner surface of the matrix helps to concentrate the electric current directly on the sample, improving the uniformity of heating and reducing heat loss. This is especially important when sintering powders with different electrical conductivity, such as Ni, Al_2_O_3_, and 3Y-ZrO_2_, since this method ensures effective sintering even when complex compositions are used in the production of WC composites [114,115].

Eith the rapid development of SPS technology, WC without a binder phase was successfully sintered and investigated via the SPS method [116]. Compared with the LPS method, the hardness of WC sintered using SPS was significantly increased, and the sintering time was also greatly reduced, but the impact toughness was seriously reduced. Zhao et al. [117] reported that sintered WC with a relative density of 99.5% and a Vickers hardness of 2414 kg·mm^−2^ was obtained by sintering at 1500 °C without soaking time, but the grain size increased from nanosized to microsized. In addition, the presence of brittle subcarbide W_2_C phase was found in the sintered products, which reduced the fracture toughness and WC hardness (since the W_2_C phase had a Vickers hardness of 17.1 kg·m^−2^ and a fracture toughness of 3.6 MPa·m^1/2^). By adding free carbon to the starting material, the influence of W_2_C phase can be reduced, and a pure sintered WC sample can be obtained.

But it should be considered that the addition of carbon creates two effects that act opposite to each other with respect to mechanical properties. The addition of carbon reduces or eliminates the brittle W_2_C phase, and this improves the fracture toughness, but carbon promotes abnormal grain growth, and this secondarily reduces the fracture toughness of WC [118].

Nominally stoichiometric WC powders with a grain size in the range of 40–70 nm can be compacted to near theoretical density (99.1%) with a grain size of 305 nm when heated at high speed to 1750 °C without soaking time. However, the sintered material contained W_2_C, and the effect of carbon addition on the presence of this phase was investigated using these powders and powders with a grain size of 12 nm. The effect of carbon on anomalous grain growth (AGG) as a function of temperature and carbon addition was investigated. The effect of heating rate to sintering temperature and holding time at this temperature was also investigated [119].

The effect of SPS process on the density, microstructure, mechanical properties, and fracture morphology of sintered gold was studied for a WC-6Co-1.5Al ball powder. The results show that with the following process parameters: base value of pulse current 360 A, peak value of 3000 A, frequency of 50 Hz, duty cycle of 50%, direct current of 1500 A, total sintering time of 6 min, and sintering pressure of 30 MPa, a density, hardness, and flexural strength of 14.2 g/cm^3^, 94HRA, and 1 can be obtained after sintering with pulse current for 1 min followed by sintering with a direct current for 5 min. An MPa of 660 is an ultrafine-grained bulk material of cemented WC-6Co-1.5Al carbide with a density of 660 MPa; With a further increase or decrease in the pulse current sintering time, the density, hardness, and bending strength of the sintered product decrease [120].

The IPS of plasma-chemical nanopowders of WC-(0.3; 0.6; 1) wt.% Co has peculiarities. The IPS process of solid composites with ultra-low cobalt content can be consistently represented as a change in the following stages: the rearrangement of particles at lower temperatures (stage I) → sintering of WC-Co particles due to creep of Co γ phase of cobalt, the intensity of which is determined by the diffusion rate along the grain boundary (stage II) → sintering due to diffusion creep, which rate is limited by volume diffusion in cobalt (stage III-1) → sintering of tungsten carbide particles along WC/WC grain boundaries under conditions of intensive grain growth (stage III-2). Samples with a high density (96.4–98.4%) and high mechanical properties (for WC-0.3% Co carbide: HV ~ 20.5 GPa, K_1_C = 7.1 MPa m^1/2^) were obtained [121].

**Figure 12:**
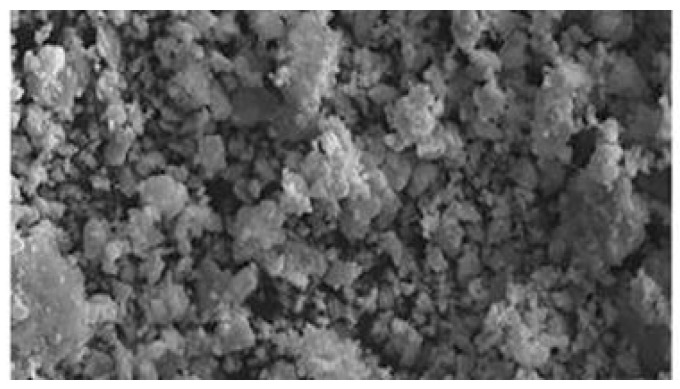
SEM image of WC-Co powder [122].

According to Alymov M. et al. [122], the possibilities and prospects of the chemical-metallurgical method and self-propagating high-temperature synthesis for obtaining submicron and nanoscale carbide powders, which form the basis of modern high-performance materials and products, are discussed. SEM images of these powders are presented in Figure 12. Specific examples of the use of submicron and nanoscale powders for obtaining tungsten-based heavy metal composites (TVS grade) and tungsten carbide-based solid composites (WC grade and its variants) for various purposes are also presented.

According to Song X et al. [123], WC-Fe-Ni-Nb and conventional WC-Co were prepared via the SPS method. The prepared powders were placed in a cylindrical graphite matrix. Then, sintering was carried out using an SPS setup. (Dr Sinter SPS 211 LX). The sintering temperatures used for WC-Fe-Ni-Nb consolidation were 1100 °C, 1200 °C, and 1300 °C. The SPS displacement curves obtained from the equipment provide information on how the sintering process promoted consolidation in each temperature range. From this information, the shrinkage rates of the sintered specimens were calculated. Densities are determined using Archimedes’ law using water as the immersion medium by measuring dry and immersed masses. Relative densities are presented as a percentage of the density calculated using the Archimedes’ method to the theoretical density estimated using the mixing rule.

More readily available elements with similar effects are Ni, Cr, Cu, Mo, Al, Si, and Fe. SPS is a synthesis and processing technology that allows for the sintering and bonding of agglomerate at low temperatures and in short time intervals via discharge between energetic particle surfaces and/or secondary discharge using Joule heating. By applying continuous on–off switching of high-power, pulsed direct current at low voltage with a spark arrester, it effectively affects the high-temperature spark plasma generated in the initial stage of short-term switching on and the electromagnetic field via pulsed direct current [124,125].

Along with this, this sintering process is characterized by energy efficiency and high densification performance and is also characterized by low energy consumption (from one-fifth to one-third compared to conventional sintering methods) such as pressure less sintering (PLS), hot pressing (HP), and hot isostatic pressing (HIP). Externally, the system resembles a standard hot pressing apparatus, but without the external heating element. However, the SPS method shows excellent results: structural adaptation, minimal grain growth, increased electrification, and a pronounced preferential orientation effect.

The SPS method is sometimes referred to as pressure pulse sintering, electric current sintering (ECAS), pulsed electric current sintering (PECS), or FAST. ECAS covers a wide range of processing methods using electric current. SPS is part of this group, but currently, the most popular technical term worldwide is SPS or SPS process, so in this paper, we generally use the term “SPS”. It is well known that SPS is an advanced processing technology for the production of homogeneous, high-density, nanostructured sintered compact materials with functional properties (FGMs), fine ceramics, composite materials, novel wear-resistant materials, thermoelectric semiconductors, and biomaterials [126].

The SPS process is a dynamic non-equilibrium treatment whose phenomenon varies from the early stage, to the middle stage, and to late stage regarding the sintering mechanism, together with the characteristics of the reacting material. Despite years of research work by many materials researchers concerning the SPS mechanism, the SPS effect, in other words, the effect of pulsed strong current on spark plasma generation and the special properties of consolidated materials, remains unclear [127,128,129].

This synthesis method was used in this work to sinter a WC-6.4Fe-3.6Ni composite. SPS was carried out on an SPS 211 LX (Dr. Sinter LAB) at temperatures of 1100, 1200, and 1300 °C under pressure of 35 MPa and vacuum of 10–5 bar, with a heating rate of 65 °C/min to synthesize solid WC-6.4Fe-3.6Ni composites. The control WC-Co composite was prepared at 1200 °C. The sintered samples were held at the maximum temperature for 5 min and cooled to room temperature for 40 min in a sintering chamber. The samples were sintered in a graphite mold, resulting in a cylindrical geometry with approximate dimensions of 6 mm (diameter) and 5 mm (height). Heating was performed with a constant, pulsed current of 20.0 ms duration. The maximum achieved current was 366 A, and the voltage was 7.6 V.

**Figure 13:**
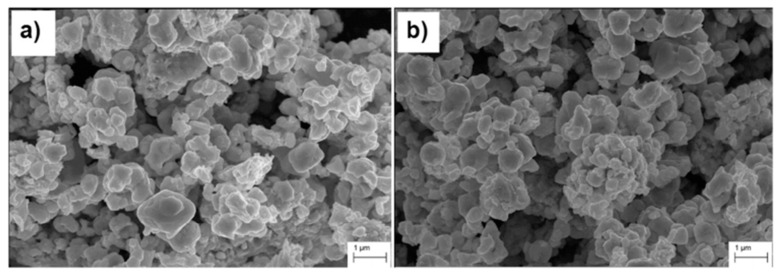
SEM images of (**a**) WC-Co and (**b**) WC-6.4Fe-3.6Ni composite powders [130].

SPS was used in [130] to synthesize the WC-8Ni-8Fe composite, focusing on obtaining a high-quality, dense material using low-cost precursors. Tungsten (VI) oxide, magnesium, carbon, nickel, and iron were used as the starting materials. Figure 13 shows the SEM image of the materials mentioned above. The sintering process was carried out at temperatures of 1000–1200 °C under a pressure of 57.3 MPa, with heating rates of 50° C/min and 87.5 °C/min. The optimum sintering temperature of 1200 °C resulted in a uniform distribution of the Ni-Fe phase and the following improved mechanical properties: a hardness of 1303 HV, a fracture toughness of 12.4 MPa, and a flexural strength of 1365 MPa. The composite showed a decrease in porosity from 13% to 0%, reaching full density. This method is suitable for industrial applications due to its cost-effective materials and high productivity.

WC-Co powder mixtures were sintered at 1200 °C, which was considered a satisfactory temperature as shown in previous works [131] related to SPS WC-10% wt. Co. Both composites were processed under the following parameters: a heating rate of 65 °C/min, a pressure of 40 MPa, a dwell time of 5 min, and a vacuum atmosphere of 102 Pa.

The processing temperature of the composites depends significantly on the type of binder. For example, the use of iron (Fe)-based binders requires higher sintering temperatures (around 1200–1400 °C) due to their high melting point and the need to ensure sufficient material density. Nickel (Ni) has a lower melting point, which allows for the reducing of the processing temperature to 1000–1200 °C, while ensuring good adhesion between particles and the formation of a strong matrix. For cobalt (Co), traditionally used as a binder, the processing temperature varies in the range of 1150–1250 °C, ensuring an optimal balance between the density, hardness, and impact toughness. Changing the binder composition, for example, adding alloying elements (Cr, Mo), can additionally affect phase transformations and sintering temperature, which requires the careful optimization of the processing parameters to achieve the desired mechanical properties [132].

SPS shows significant advantages for the synthesis of cobalt-free composites in which cobalt is replaced by nickel and iron. First of all, SPS allows for the effective control of the material microstructure, ensuring a uniform distribution of nickel and iron in the WC matrix. This improves phase adhesion and enhances mechanical properties such as strength and wear resistance [133]. Due to the low temperatures and short sintering cycles, SPS minimizes the risk of porosity and grain growth, which is particularly important for maintaining high hardness and durability of cobalt-free composites. The use of SPS also enhances corrosion resistance due to the uniform distribution of iron and nickel, which makes such composites more resistant in corrosive environments compared to conventional WC-Co composites [134]. As a result, SPS makes it possible to create high-performance cobalt-free composites with optimized properties suitable for applications in various high-tech industries such as aerospace, energy, and mechanical engineering.

The development of material-processing technologies and the creation of new alloys in the near future will be aimed at improving performance, cost effectiveness, and environmental safety, which includes replacing cobalt (Co) with more affordable and environmentally sustainable materials such as iron (Fe), nickel (Ni), intermetallics (TiAl, TiCuAl), and high-entropy alloys (HEAs) with a balanced combination of strength, heat resistance, and corrosion resistance. The use of these materials requires further research into their behavior under the operating conditions and the adaptation of the processing technologies, including additive manufacturing (SLM and BJAM), which allows for the creation of complex shapes at minimal cost, and spark plasma sintering (SPS), which reduces the processing time and improves the microstructure. The integration of these advanced methods with digital manufacturing process control technologies promises to be a key stage in the technological revolution, providing flexibility, efficiency, and competitiveness in various industries.

The highest-volume production of WC + Co cermets is primarily carried out through powder metallurgy techniques such as sintering, hot pressing, and infiltration. These methods allow for the efficient consolidation of tungsten carbide particles with cobalt binders into dense, homogenous materials that exhibit exceptional hardness, wear resistance, and toughness. Major production hubs are concentrated in regions with well-established metallurgical industries, including China, Europe, and North America.

To scale these processes and compete with high bulk fabrication, modern approaches are focusing on advanced sintering techniques like SPS and FAST. These methods enable faster cycle times and energy-efficient production while maintaining the material’s properties. Hybrid manufacturing techniques that integrate traditional powder metallurgy with additive manufacturing (AM) are also emerging, allowing for the production of complex geometries and reducing material waste.

The automation of production lines and the integration of real-time monitoring systems further enhance scalability and consistency in industrial settings. Additionally, advancements in binder technology, such as the use of cobalt-free alternatives or high-entropy alloys, open new pathways for sustainable and cost-effective production.

By combining innovation in processing techniques, material optimization, and production automation, these modern methods hold the potential to establish themselves as viable alternatives to traditional high-volume fabrication, meeting the growing industrial demand for WC + Co cermets.

## 6. Conclusions

Thus, it should be noted that the studies highlight the importance of developing cobalt-free WC-based solid composites as a promising direction in materials science and industrial applications. Conventional WC-Co composites, despite their excellent mechanical properties, face the problems of high cost, cobalt scarcity, and environmental risks. Replacing cobalt with more affordable and environmentally friendly materials such as nickel and iron provides an effective solution that not only maintains but also improves key composite performance including strength, wear resistance, and corrosion resistance. Synthesis techniques such as powder metallurgy, encapsulation, 3D printing, and especially SPS have demonstrated high efficiency in creating composites with optimized microstructure and minimal grain growth. These technologies produce composites with high density, excellent mechanical properties, and resistance to aggressive environments, making them attractive for use in engineering, metallurgy, mining, and aerospace applications. Cobalt-free WC-based composites using nickel and iron as binders not only match the performance of WC-Co materials but also outperform them in a number of parameters, making them a key element in the future development of environmentally friendly and cost-effective solutions for industrial needs.

## Figures and Tables

**Table 1:** Additive manufacturing techniques used for printing WC-Co hard metals [94].

AM Process	Abbreviation	Other Names	Advantages	Disadvantages
Selective laser melting	SLM	Laser powder bed fusion (L-PBF)	High dimensional accuracy High geometric freedom Less steps High hardness	High residual stress Uneven microstructure Carbon loss and evaporation of Co
Selective electron beam melting	SEBM	Electron beam powder bed fusion (E-PBF)	High dimensional accuracy High geometric freedom Less steps High hardness High scan speed	High residual stress Uneven microstructure Expensive equipment Needs vacuum
Binder jet additive manufacturing	BJAM	Binder jet 3D printing (BJ3DP)	Uniform microstructure High toughness Low cost Low residual stress	Complicated processes Large shrinkage Low hardness, moderate strength
3D gel-printing	3DGP	N/A	Low residual stress Uniform microstructure Low powder requirements No raw material loss	Complicated processes Large shrinkage
Fused filament fabrication	FFF	N/A	Low residual stress Uniform microstructure Low powder requirements No raw material loss	Complicated processes Large shrinkage Needs filament fabrication equipment Rough surface

**Table 2:** Typical mechanical properties of fine WC/Co hard alloys sintered via SPS [102].

Product Code Name	Co Content wt%	WC pdr. Grain Size μm	Density g/cm^3^	Hardness mHv	Transverse Rupture Strength MPa	Fracture Toughness K_1_C
TC-05	<2	<0.5	15.2	2350	2300	6.2
TC-10	<4	<0.5	15.0	2150	2640	6.5
TC-20	<6	<0.5	14.8	2050	2940	7.3
M78	0	<0.2	15.4	2600	1500	5.1
WC100	0	<0.08	15.6	2700	1470	5.6
NC100	0	<0.5	15.4	2570	1180	5.4

## Data Availability

Data are contained within the article.
